# Anti-inflammatory effect of semaglutide: updated systematic review and meta-analysis

**DOI:** 10.3389/fcvm.2024.1379189

**Published:** 2024-07-05

**Authors:** Walter Masson, Martín Lobo, Juan Patricio Nogueira, Alfredo Matias Rodriguez-Granillo, Leandro Ezequiel Barbagelata, Daniel Siniawski

**Affiliations:** ^1^ Department of Cardiology, Hospital Italiano de Buenos Aires, Buenos Aires, Argentina; ^2^ Department of Cardiology, Hospital Militar Campo de Mayo, Buenos Aires, Argentina; ^3^ Endocrinology, Nutrition and Metabolism Research Center, Faculty of Health Sciences, Universidad Nacional de Formosa, Formosa, Argentina; ^4^ Medicine and Surgery Department, Universidad Internacional de las Américas, San José, Costa Rica; ^5^ Clinical Research Department, Centro de Estudios en Cardiologia Intervencionista (CECI), Buenos Aires, Argentina; ^6^ Department of Interventional Cardiology, Sanatorio Otamendi, Buenos Aires, Argentina

**Keywords:** semaglutide, inflammation, C-reactive protein, glucagon-like peptide-1 receptor agonists, meta-analysis

## Abstract

**Background:**

The anti-inflammatory effect could be one of the mechanisms by which semaglutide reduces cardiovascular risk in patients with type 2 diabetes mellitus (T2DM) and/or obesity. Determining the anti-inflammatory effect of semaglutide was the objective of this systematic review and meta-analysis.

**Methods:**

This meta-analysis was performed according to the PRISMA guidelines. A literature search was performed to detect randomised clinical trials that have quantified the effect of semaglutide on C-reactive protein (CRP) levels compared to placebo or a control group (other glucose-lowering drugs). The primary outcome was CRP index (final CRP/basal CRP). A random-effects model was used.

**Results:**

Thirteen randomised clinical trials were considered eligible (*n* = 26,131). Overall, semaglutide therapy was associated with lower CRP index values compared to the placebo group (SMD −0.56; 95% CI −0.69 to −0.43, *I*^2^ 92%) or the control group (SMD −0.45; 95% CI −0.68 to −0.23, *I*^2^ 82%).Such an association was similarly observed when different treatment regimens (subcutaneous vs. oral) or different populations (patients with or without T2DM) were analysed. The sensitivity analysis showed that the results were robust.

**Conclusion:**

The present meta-analysis demonstrated that the use of semaglutide was associated with a reduction in inflammation irrespective of the population evaluated or the treatment regimen used. These findings would explain one of the mechanisms by which semaglutide reduces cardiovascular events.

**Systematic Review Registration:**

PROSPERO [CRD42024500551].

## 1 Introduction

Glucagon-like peptide-1 (GLP-1RA) receptor agonists possess multiple favorable metabolic, anti-inflammatory effects and their use is associated with marked body weight reduction (1–3). More importantly, this drug class has been shown to reduce cardiovascular events in patients with type 2 diabetes mellitus (T2DM) at high cardiovascular risk or with established cardiovascular disease (4, 5). Accordingly, current guidelines recommend the use of GLP-1RAs as first-line antidiabetic therapies in patients at high cardiovascular risk (6).

Semaglutide is a potent GLP-1RA used in the treatment of T2DM with proven cardiovascular benefits (7). It is currently available in formulations for oral and subcutaneous administration (8). In addition, the use of higher doses of semaglutide was associated with a marked decrease in body weight in patients with overweight or obesity (9). Recently, the cardiovascular benefit with the use of high doses of semaglutide was also seen in patients with overweight or obesity and high cardiovascular risk but without T2DM (10).

Multiple mechanisms have been proposed to explain the cardiovascular benefit of semaglutide. These include direct cardiac effects (protection against myocardial ischaemia, reduction of epicardial adipose tissue and improvement of cardiac contractility), vascular (vasodilator effect and improvement of endothelial dysfunction), renal (reduced glomerular filtration rate and proteinuria drop) and metabolic (blood pressure decreased, lipid profile improved and glucose-lowering effect) (11).

Inflammation plays a crucial role in the development and progression of atherosclerosis and its cardiovascular complications (12). Likewise, elevation of inflammatory markers predicts the development of T2DM and its complications (13–15). Strong evidence has shown the anti-inflammatory effect of GLP-1RA (16, 17). The favorable effects on atherogenic lipoproteins and hepatic steatosis indices support the pleiotropic benefits of semaglutide beyond glycemic control (18). Consequently, anti-inflammatory actions could be an additional relevant mechanism to explain the cardiovascular benefit of this type of drugs. A previously published meta-analysis has reported a significant decrease in several anti-inflammatory markers with the use of GLP-1RA (19). However, it did not include trials with semaglutide.

Considering what has previously been discussed, the primary objective of this study was to perform a systematic review and updated meta-analysis on the anti-inflammatory effect of semaglutide.

## 2 Material and methods

### 2.1 Data extraction and quality assessment

This study was conducted in accordance with the PRISMA guidelines (Preferred Reporting Items for Systematic Reviews and Meta-Analyses) to report systematic reviews (20) (PRISMA checklist in Supplementary Material). This systematic review was recorded in PROSPERO (CRD42024500551).

A literature search was performed identifying clinical trials of semaglutide published until 01 Dec 2023. As two independent reviewers searched the electronic PubMed/MEDLINE, Scielo, Embase and Lilacs databases using the either the Medical Subject Headings (MeSH) terms or keywords “semaglutide” or “GLP-1RA”, combined with “inflammation” or “C-reactive protein (CRP)” or “cytokines”, or using the term “semaglutide” with the “randomised clinical trials” filter, and the data were extracted. To match each individual descriptor and define the search, we use the Boolean operator “AND.” In addition, the authors also searched for ’snow ball’ to find other articles of interest. Only studies conducted in humans were included No language restrictions were used in the search.

The following inclusion criteria were established: (a) Studies that have analysed the anti-inflammatory effect of semaglutide (expressed as ultra-sensitive blood CRP levels) compared to a placebo group or a group with another anti-diabetic regimen; (b) Studies with a duration of follow-up ≥3 months; (c) Randomized clinical trials.

Potential risks of bias were evaluated for all included trials, using a tool developed for this purpose (Rob 2) (21). This tool assesses bias on five different domains: bias arising from randomisation, bias due to deviations from planned intervention, bias due to lack of outcome data, bias in outcome measurement, and bias in selection of reported outcome. Each domain was rated as “high risk”, “low risk” or “with some concerns”, further obtaining an overall rating of each study. Two authors determined the risk of bias for each article. Any disagreement was resolved with a third reviewer.

### 2.2 Statistical analysis

The effect of semaglutide therapy on CRP reduction was calculated. Effect size measures were expressed as standardised mean differences (SMDs) between CRP indices with their respective 95% confidence intervals (95% CIs). These indices were obtained by dividing the final value by the baseline CRP value into both groups. The 95% CIs were calculated manually when not reported in the original publications (22). Furthermore, statistical *I*^2^ was calculated to quantify heterogeneity and inconsistency between studies. A random-effects model was chosen because the trials differ in the populations included or in the follow-up time and because the calculated heterogeneity (*I*^2^) was elevated. To compare the average effect between subgroups, we used a Z-test. The level of statistical significance was set to 0.05 (2-tail analysis). Statistical software R (version 3.5.1) was used for the analysis (23).

### 2.3 Sensitivity analysis

The sensitivity analysis consists of replicating the results of the meta-analysis, excluding at each step, each of the studies included in the review. If the results obtained are similar, both in the direction/magnitude of the effect and in the statistical significance, the analysis indicate that the result is robust.

### 2.4 Analysis of publication bias

Begg & Mazumdar test and the Egger test with mixed effects model were done. Additionally, a funnel plot using the standard error for standardized mean difference was created.

## 3 Results

### 3.1 Characteristics of the included studies

**Figure 1:**
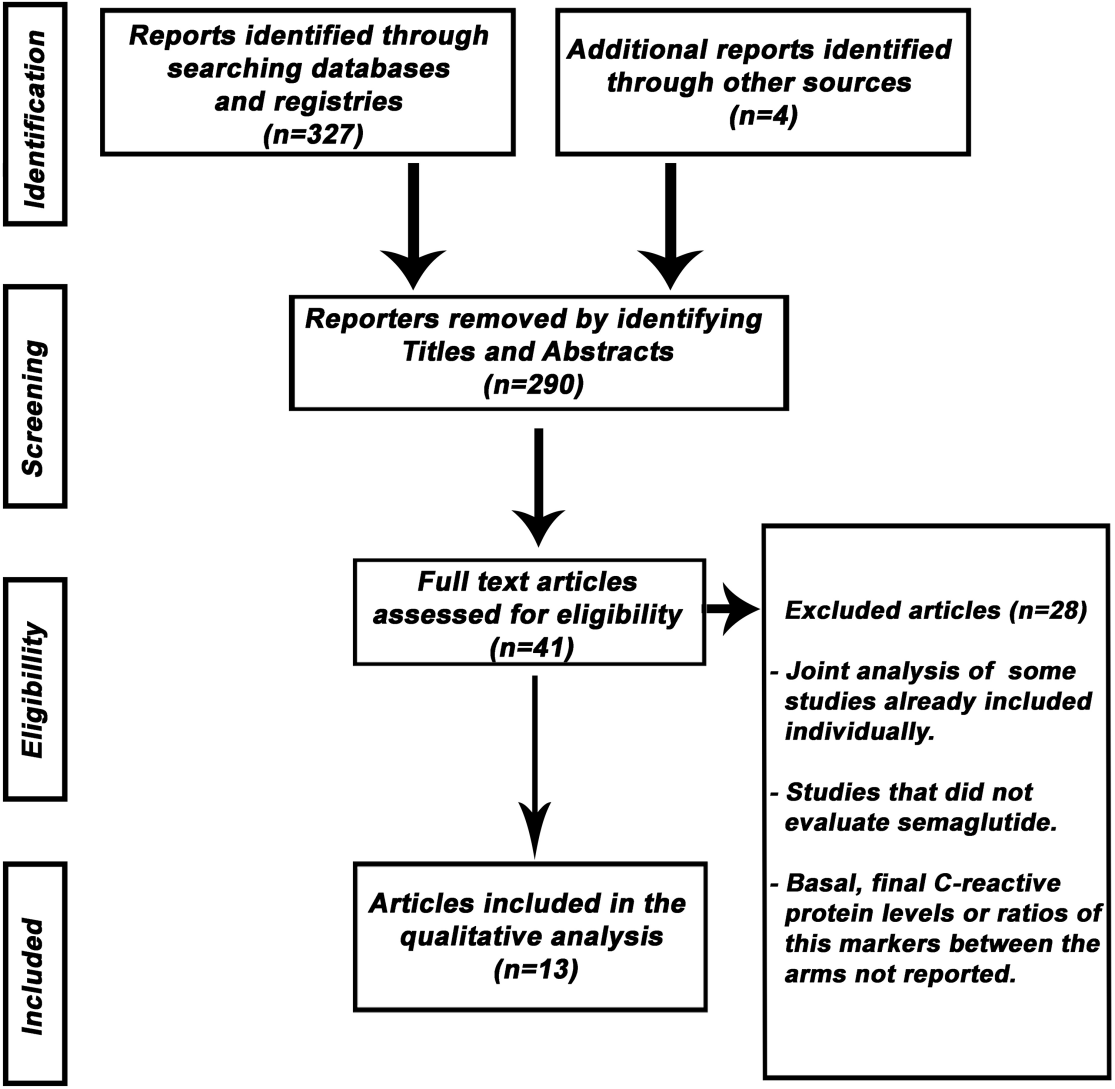
Flow diagram of the study selection process.

The search included 331 potentially relevant articles after examining the titles/summary, excluding 290 studies because they were duplicate publications or because they did not evaluate the purpose of this study. After careful reading of the remaining articles, 29 studies were removed, because the exposure/event of interest was not reported. A flowchart of the trial selection process is shown in Figure 1.

Thirteen randomised clinical trials (*n* = 26,131 patients) were identified and considered eligible for this systematic review (10, 24–35). A total of 13,923 subjects were allocated to the semaglutide group and 12,208 individuals were randomised to a control group. Such a control group was assigned to placebo in 10 trials (10, 24, 27–32, 34, 35), to another anti- diabetic drug in 2 trials (exenatide and empagliflozin) (24, 26) and to both options in the remaining trial (liraglutide and placebo) (33).

In total, 5 studies included patients with T2DM (24–27, 29) while another 6 studies evaluated patients with overweight or obesity without T2DM (10, 28, 30, 31, 33, 34). Within the latter, a study specifically including patients with heart failure with preserved ejection fraction is highlighted (35). Finally, a study simultaneously analysed patients with or without T2DM (32). The population characteristics of the studies included in this review are shown in Table 1.

**Table 1:** Characteristics of trials evaluated in the meta-analysis.

Study (year)	*N*	Semaglutide scheme	Comparator	Population	Follow-up
SUSTAIN-3 (24)	806	1 mg SC × week	Exenatide 2 mg SC × week	Patients ≥18 years with T2DM (HbA_1c_ 7.0%–10.5%) on one or two stable treatments for T2DM. Mean HbA_1c_: 8.3%; mean BMI: 33.8 kg/m^2^; females: 44.7%; mean age: 56.6 years.	56 weeks
PIONEER-1 (25)	703	3, 7 and 14 mg PO × day	Placebo	Patients ≥18 years with T2DM (HbA_1c_ 7.0%–9.5%), managed with diet and exercise only. Mean HbA_1c_: 8%; mean BMI: 31.8 kg/m^2^; females: 49.2%; mean age: 55 years.	26 weeks
PIONEER-2 (26)	821	14 mg PO × day	Empagliflozin 25 mg PO	Patients ≥18 years with T2DM (HbA_1c_ 7.0%–10.5%) on stable doses of metformin. Mean HbA_1c_: 8.1%; mean BMI: 32.8 kg/m^2^; females: 49.5%; mean age: 58 years.	52 weeks
PIONEER-5 (27)	324	14 mg PO × day	Placebo	Patients ≥18 years with T2DM (HbA_1c_ 7.0%–9.5%), with a GFR between 30 and 59 ml/min/1.73 m^2^, receiving stable treatment for T2DM. Mean HbA_1c_: 8%; mean BMI: 32.4 kg/m^2^; females: 52%; mean age: 70 years.	26 weeks
STEP-1 (28)	1,961	2.4 mg SC × week	Placebo	Adults with a BMI ≥ 30 or ≥27 kg/m^2^ with 1 or more weight-related conditions (hypertension, dyslipidaemia, OSA. CVD), without T2DM. Mean BMI: 37.9 kg/m^2^; females: 74.1%; mean age: 46 years; prediabetes 43.7%.	68 weeks
STEP-2 (29)	1,132	1 and 2.4 mg SC × week	Placebo	Adults with a BMI ≥ 27 kg/m^2^ and HbA_1c_ between 7 and 10% who had been diagnosed with T2DM within 180 days prior to assessment. Mean HbA_1c_: 8.1%; mean BMI: 35.7 kg/m^2^; females: 50.9%; mean age: 55 years.	68 weeks
STEP-3 (30)	604	2.4 mg SC × week	Placebo	Adults with a BMI ≥ 30 or ≥27 kg/m^2^ with 1 or more weight-related conditions (hypertension, dyslipidaemia, OSA. CVD), without T2DM. Mean BMI: 38 kg/m^2^; females: 81%; mean age: 46 years.	68 weeks
STEP-5 (31)	304	2.4 mg SC × week	Placebo	Adults with a BMI ≥ 30 or ≥27 kg/m^2^ with 1 or more weight-related conditions (hypertension, dyslipidaemia, OSA. CVD), without T2DM. Mean BMI: 38.5 kg/m^2^; females: 77.6%; mean age: 47.3 years.	104 weeks
STEP-6 (32)	391	1.7 and 2.4 mg SC × week	Placebo	Adults from Japan and South Korea with a BMI ≥ 27 kg/m^2^ with 2 or more weight-related conditions [hypertension, dyslipidaemia, T2DM (Japan only)], or ≥35 with 1 or more associated conditions. Mean BMI: 31.9 kg/m^2^; females: 37%; mean age: 51 years; T2DM: 25%.	68 weeks
STEP-8 (33)	243	2.4 mg SC × week	Liraglutide 3 mg SC × day and placebo	Adults with a BMI ≥ 30 or ≥27 kg/m^2^ with 1 or more weight-related conditions (hypertension, dyslipidaemia, OSA. CVD), without T2DM. Mean BMI: 37.5 kg/m^2^; females: 78.4%; mean age: 49 years.	68 weeks
OASIS-1 (34)	608	50 mg PO × day	Placebo	Adults with a BMI ≥ 30 or ≥27 kg/m^2^ with 1 or more weight-related conditions (hypertension, dyslipidaemia, OSA. CVD), without T2DM. Mean BMI: 37.5 kg/m^2^; females: 73%; mean age: 50 years.	68 weeks
SELECT (10)	17,604	2.4 mg SC × week	Placebo	Patients ≥45 years with established CVD, a BMI ≥ 27 kg/m^2^ and without TDM2. Mean BMI: 33.3 kg/m^2^; females: 27.7%; mean age: 61.6 years.	159.2 weeks
STEP-HfpEF (35)	529	2.4 mg SC × week	Placebo	Patients ≥18 years with a BMI ≥ 30 kg/m^2^ and heart failure with preserved ejection fraction (≥45%), without T2DM. BMI (median): 37 kg/m^2^; females: 56.1%; age (median): 69 years.	52 weeks

BMI, body mass index; CVD, cardiovascular disease; HTN, arterial hypertension; GFG, glomerular filtration rate; OSA, obstructive sleep apnoea; PO, orally; SC, subcutaneous; T2DM, type 2 diabetes mellitus.

### 3.2 Quality evaluation of included studies

**Figure 2:**
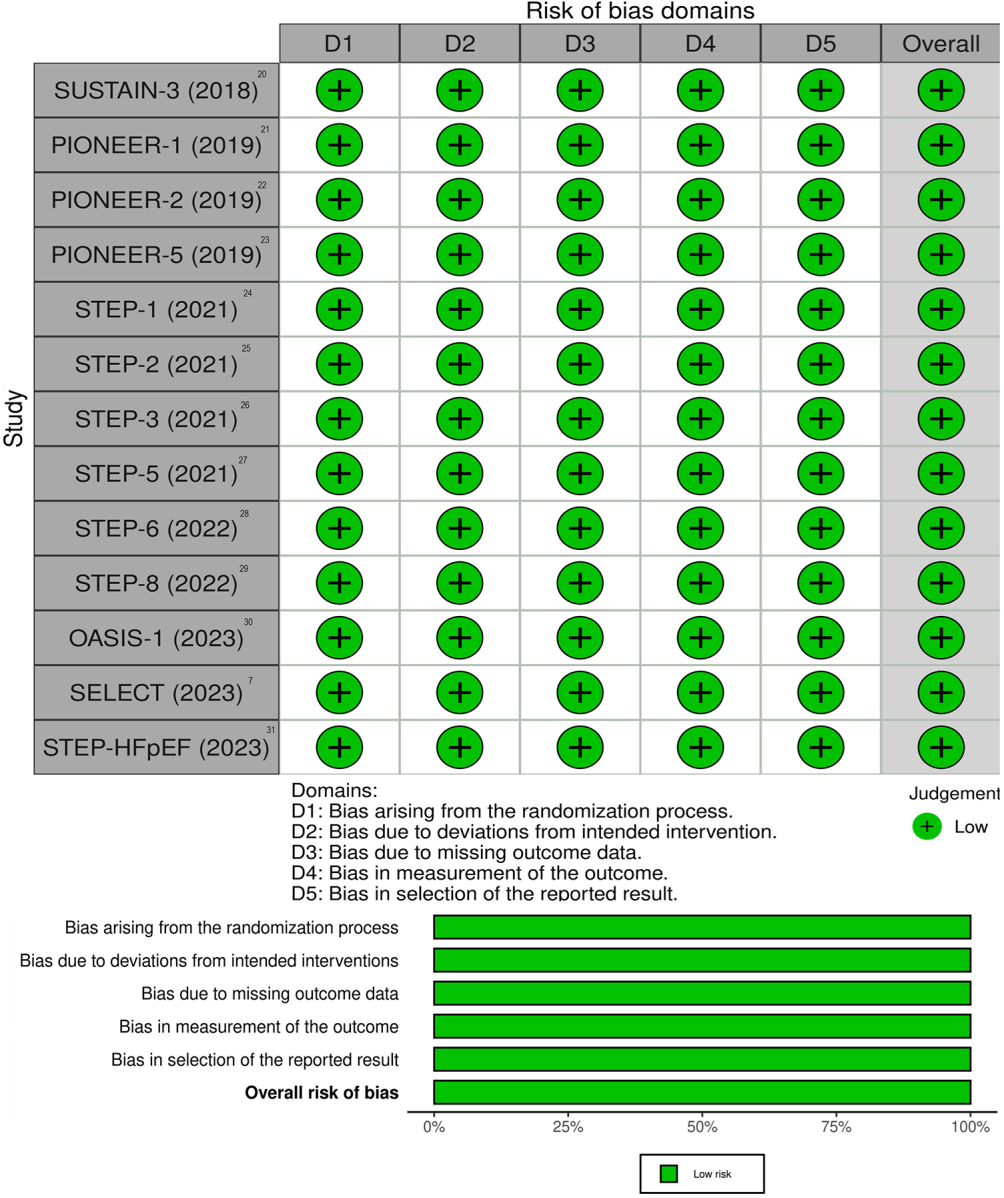
Summary and evaluation of individual bias of included studies.

All studies included in this review showed low risk of bias. The quality of the selected studies is shown in Figure 2.

### 3.3 Impact of semaglutide on inflammatory markers

**Figure 3:**
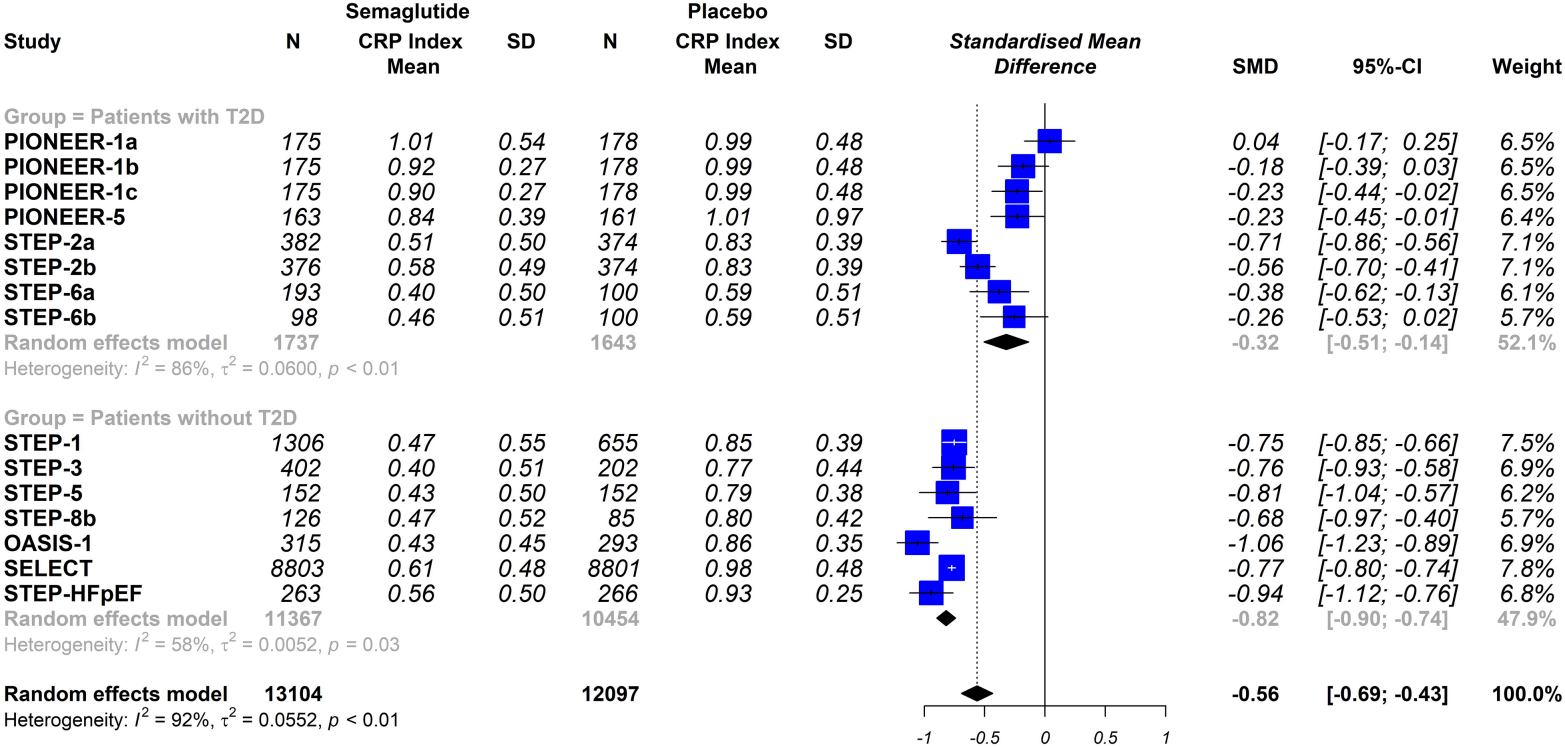
Effect of semaglutide therapy on CRP index. Random-effects model, standardised mean differences (SMD), 95% confidence intervals (CI) and statistical *I*^2^. Top: semaglutide vs. placebo trial group. Bottom: group of studies with semaglitide versus other drugs. PIONEER-1 a, b and c: semaglutide 3, 7 and 14 mg orally, respectively. STEP-2 a and b: semaglutide 2.4 mg and 1 mg subcutaneous, respectively. STEP-6 a and b: semaglutide 2.4 mg and 1.7 mg subcutaneously, respectively. STEP-8b: semaglutide 2.4 mg arm vs placebo.

Overall, this meta-analysis shows that semaglutide therapy was associated with lower CRP index values compared to placebo (SMD −0.56; 95% CI −0.69 to −0.43, *I*^2^ 92%) or when comparing semaglutide treatment vs. a control group consisting of patients medicated with other glucose-lowering drugs (SMD −0.45; 95% CI −0.68 to −0.23, I 382%) (Figure 3).

**Figure 4:**
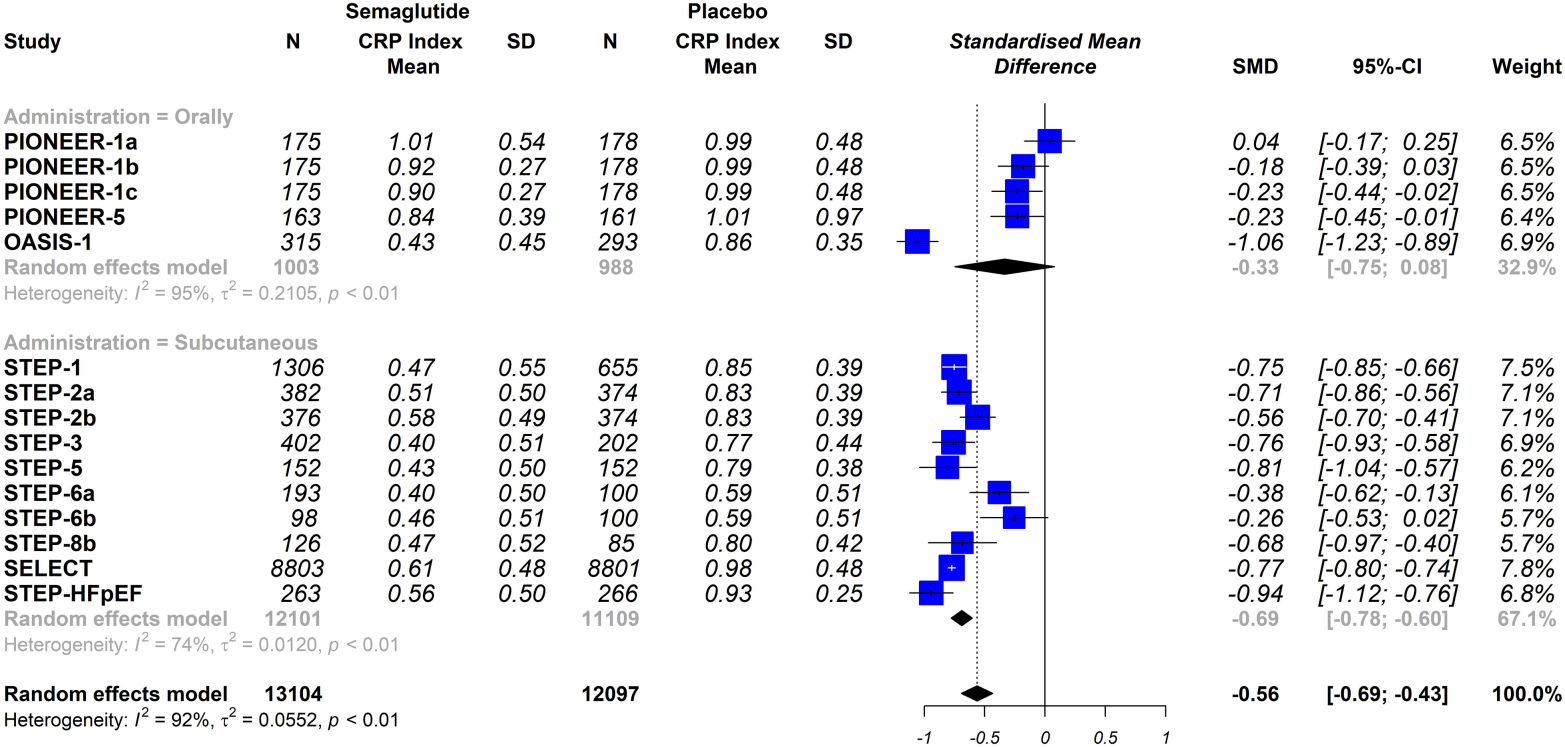
Effect of semaglutide therapy on CRP rate stratified by groups of patients with or without type 2 diabetes mellitus (T2DM). Analysis performed only with trials vs. placebo. Random-effects model, standardised mean differences (SMD), 95% confidence intervals (CI) and statistical *I*^2^. PIONEER-1 a, b and c: semaglutide 3, 7 and 14 mg orally, respectively. STEP-2 a and b: semaglutide 2.4 mg and 1 mg subcutaneous, respectively. STEP-6 a and b: semaglutide 2.4 mg and 1.7 mg subcutaneously, respectively. STEP-8b: semaglutide 2.4 mg arm vs placebo.

**Figure 5:**
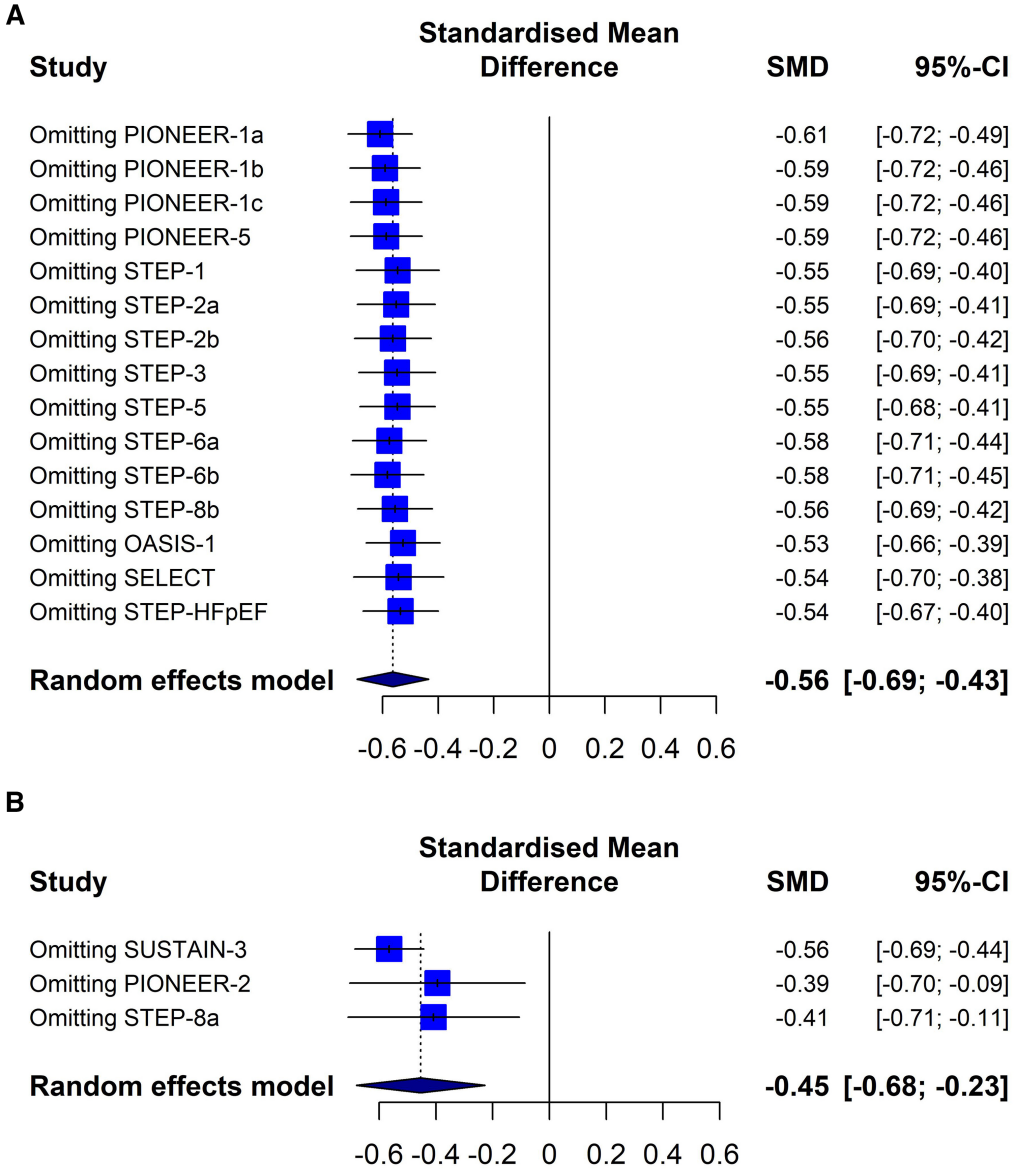
The effect of semaglutide therapy on the stratified CRP rate by the way semaglutide was administered in diabetic (**A**) and non-diabetic population (**B**). Analysis performed only with trials vs. placebo. Random-effects model, standardised mean differences (SMD), 95% confidence intervals (CI) and statistical *I*^2^. PIONEER-1 a, b and c: semaglutide 3, 7 and 14 mg orally, respectively. STEP-2 a and b: semaglutide 2.4 mg and 1 mg subcutaneous, respectively. STEP-6 a and b: semaglutide 2.4 mg and 1.7 mg subcutaneously, respectively. STEP-8b: semaglutide 2.4 mg arm vs. placebo.

The stratified analysis was performed by analysing only the trials that used a placebo group as comparator, When the trials were analysed according to the different dosing schemes (subcutaneous vs. oral), the results were not statistically significantly different (oral semaglutide group: SMD −0.33; 95% CI −0.75 to 0.08, *I*^2^ 95%); semaglutide subcutaneous dose group: SMD −0.69; 95% CI −0.78 to −0.60, *I*^2^ 74%); interaction *p* = 0.098 (Figure 4). Furthermore, when the trials were analysed according to the included populations (patients with or without T2DM), the results were similar (patients with T2DM: SMD −0.32; 95% CI −0.51 to −0.14, *I*^2^ 86%); patients without T2DM: SMD −0.82; 95% CI −0.90 to −0.74, *I*^2^ 58%); interaction *p* ≤ 0.0001 (Figure 5).

### 3.4 Publication bias and sensitivity analysis

**Figure 6:**
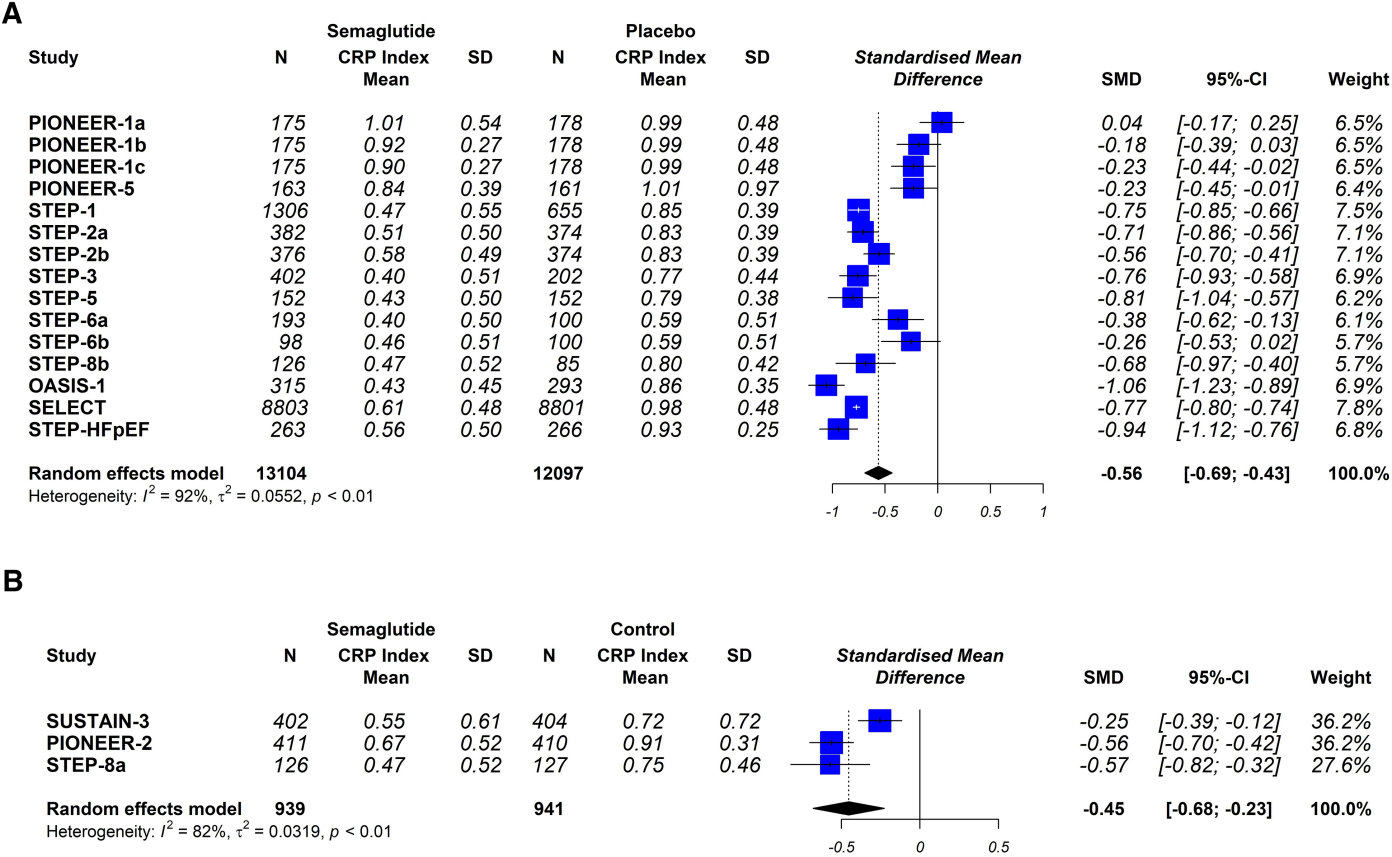
Sensitivity analysis. (**A**) Semaglutide versus placebo trial group. (**B**) Group of studies with semaglitide versus other drugs. PIONEER-1 a, b and c: semaglutide 3, 7 and 14 mg orally, respectively. STEP-2 a and b: semaglutide 2.4 mg and 1 mg subcutaneous, respectively. STEP-6 a and b: semaglutide 2.4 mg and 1.7 mg subcutaneously, respectively. STEP-8b: semaglutide 2.4 mg arm vs. placebo.

The sensitivity analysis showed the same directionality and magnitude of the overall results when the trials were excluded one by one (Figure 6). The analytical evaluation by the Begg & Mazumdar and the Egger tests do not suggest publication bias (*p* = 0.472 and *p* = 0,102, respectively). The graphic representation does not show a clear asymmetry (Supplementary Figure S1).

## 4 Discussion

In this meta-analysis we observed that treatment with semaglutide compared to placebo or to a control group that included patients medicated with other glucose-lowering drugs, was associated with lower CRP levels, regardless of the regimen used or of the population evaluated. Compared to placebo or the control group receiving other glucose-lowering drugs, the CRP rate in the semaglutide treated group was 44% and 55% lower, respectively. This value is adequately adjusted with the CRP reductions associated with the use of semaglutide observed in some of the studies included in this review (range between 39.1% and 59.6%) (10, 30, 31, 33, 35).

Evidence from epidemiological studies has shown that elevated CRP levels, a surrogate for systemic inflammation, are associated with increased cardiovascular risk (36). Furthermore, in some clinical trials evaluating patients treated with statins, elevated CRP levels were a predictor of the risk of future cardiovascular events and death stronger than cholesterol bound to low-density lipoprotein (LDL-C) itself (37). CRP is predominantly synthesised in the liver, mainly in response to interleukin-6 (IL-6) and to a lesser extent to interleukin 1β (IL-1β) and 17 (IL-17) and tumour necrosis factor alfa (TNF-α) (38). Furthermore, the involvement of inflammasomes is relevant in this process. The latter are complexes of high molecular weight proteins formed in the cytosolic compartment in response to different stimuli. Among the most widely studied in the context of atherosclerosis is the cytosolic multiprotein signaling complex called the NLRP3 inflammasome, which serves as a platform for the activation of caspase-1 and promotes the synthesis of pro-inflammatory cytokines (39).

In clinical practice, CRP values exceeding 3 mg/L are considered as a marker of cardiovascular risk (40, 41). Interestingly, 6 of the 8 studies included in this systematic review reporting baseline CRP values showed elevated levels (between 3 and 4.8 mg/L), reflecting that the populations evaluated, mostly patients with T2DM and obesity, possess a chronic inflammatory state.

Several drugs evaluated in the field of cardiovascular prevention have shown to simultaneously have an anti-inflammatory (CRP lowering) and cardioprotective effect (decrease in cardiovascular atherosclerotic events). These results strongly suggest that in addition to LDL-C, CRP could be a new treatment target. Statins, colchicine, IL1β receptor antagonists and bempedoic acid are some examples (42–45).

Furthermore, many *in vitro* studies have shown that GLP-1RAs may attenuate or suppress the expression of various inflammatory factors, including TNF-α, IL-6 and endothelial adhesion molecules (46, 47). In addition, GLP-1RAs have been found to inhibit the activation of the NLRP3 inflammasome, thereby reducing the maturation and release of inflammatory cytokines (48). Another systemic anti-inflammatory mechanism induced by liraglutide and semaglutide and involved in the reduction of CRP and cardiovascular risk, is decreased intestinal permeability through activation of Brunner's gland secretion and modulation of intraepithelial lymphocytes function (49). Moreover, GLP-1RAs appear to exert a direct epigenetic effect in patients with T2DM, regulating microRNAs that are involved in maintaining endothelial cell homeostasis (50). Accordingly, the anti-inflammatory mechanisms associated with GLP-1RA treatment could be closely linked to the cardiovascular benefit seen in clinical trials (7, 10). Furthermore, GLP-1RA therapy might play a relevant role in the treatment of other entities that possess a chronic inflammatory component, including steatohepatitis, neurodegenerative disorders, diabetic nephropathy, asthma or psoriasis (51–54).

A previously published meta-analysis showed that the use of GLP-1RA was associated with a significant reduction in CRP levels (18). However, the vast majority of the information comes from studies that have evaluated the use of exenatide and liraglutide. Furthermore, a descriptive and exploratory analysis from the PIONEER and SUSTAIN programs also showed that the use of semaglutide was associated with a reduction in CRP, although this analysis only included 4 trials (55). In this context, our meta-analysis first evaluated all information reported in clinical trials on the association between the use of semaglutide and CRP levels.

Although the initial presentation of semaglutide for clinical use was subcutaneous, administered once weekly, an oral formulationwas recently developed (56). The pharmacokinetic and pharmacodynamic differences between these formulations could be associated with different biological effects (57). In that regard, the results of our study showed that the anti-inflammatory effect was independent of the dosage form used. While the primary outcome in the subgroup of trials using oral semaglutide “rips 0”, the interaction *p*-value was not statistically significant.

Another interesting finding of this meta-analysis was that the effect on CRP levels was observed in both the population with or without T2DM. The elucidation of various cellular mechanisms linking inflammation with insulin resistance and β-cell dysfunction has revolutionised knowledge about the molecular pathogenesis of T2DM, currently establishing that this entity is a metabolic and inflammatory disorder (58). In this context, and given the link between inflammation and progression of T2DM and its complications, the finding of our study on CRP decline in this population is relevant. On the other hand, it has also been observed that patients with obesity have a chronic inflammatory state of low grade (59). In addition, obesity is considered a pre-diabetic state (60). Consequently, we would be faced with a “continuous” pathophysiology involving adipose tissue, inflammation, insulin resistance, pancreatic dysfunction, the occurrence of T2DM and development of micro- and macrovascular complications. All of this makes the findings from our study on the anti-inflammatory impact of semaglutide in the population without T2DM, mostly with obesity and pre-diabetes, of clinical relevance as well. Looking at the stratified analysis, it would appear that the impact on the CRP rate is greater in the population without T2DM, although such findings could relate to the higher semaglutide doses used in this population.

Finally, our findings showed a clear anti-inflammatory effect of semaglutide when compared to placebo, but also when compared to other anti-diabetic drugs with proven cardiovascular benefit. Interestingly, within the drugs tested were other GLP-1RAs such as exenatide or liraglutide. It would thus appear that the molecular, pharmacokinetic and pharmacodynamic differences in these drugs could influence the anti-inflammatory effect (61).

In summary, alongside its well-established anti-inflammatory properties, the impact on weight loss, glycemic control, and reduction of cardiovascular risk factors—such as blood pressure and lipid profile—explains the cardiovascular benefits of semaglutide (62, 63).

This meta-analysis has some limitations. First, there was clinical heterogeneity due to the different characteristics of the populations and the different follow-up times. Likewise, statistical heterogeneity was high. Although *I*^2^ is commonly used for assessment of heterogeneity, it is not a perfect measure and its value depends on the precision and size of the included studies. In this case, high heterogeneity can be attributed more to the magnitude of the effect than to the direction of the effect, being influenced by the low number of patients evaluated in many of the studies. In that regard, the sensitivity analysis showed robust results. Second, we were unable to quantitatively analyze the absolute or percentage reduction in CRP values because these data were not published by the majority of the original studies. Instead, we were able to analyze the index between the final and baseline values for each arm. Interpreting the index clinically proves more challenging than simply describing absolute values. Third, due to the absence of reported data in the original studies, we were unable to analyze additional inflammatory markers. Another frequently used inflammatory markers include acute-phase proteins, essentially serum amyloid A, fibrinogen and procalcitonin, and cytokines, predominantly TNFα, interleukins 1β, 6, 8, 10 and 12 and their receptors and IFNγ (64). However, their use in clinical practice is often limited, due to lacking analytical or clinical validation, or technical challenges. Four, the inability to identify additional therapies and any concomitant pathologies that could affect the inflammatory state represents another limitation of this study. Finally, the analysis of the studies comparing the use of semaglutide with other antidiabetic drugs only included a small number of studies. Therefore, additional information will be required to establish definitive conclusions.

## 5 Conclusions

The present updated meta-analysis of randomised clinical trials demonstrated that the use of semaglutide was associated with a marked anti-inflammatory effect. According to the stratified analysis, the effect would occur irrespective of the schemes used or the populations tested. The inflammatory pathway could explain much of the cardiovascular benefit seen in large clinical trials with semaglutide.

## Data Availability

The original contributions presented in the study are included in the article/Supplementary Material, further inquiries can be directed to the corresponding author.

## Supplementary material

Supplementary Figure 1 Funnel plot to assess publication bias.
