# Supplementary figures and images for: Anti-inflammatory effect of semaglutide: updated systematic review and meta-analysis
Source: Front Cardiovasc Med. 2024 Jul 5;11:1379189. doi: 10.3389/fcvm.2024.1379189 (PMC11270812; doi:10.3389/fcvm.2024.1379189)

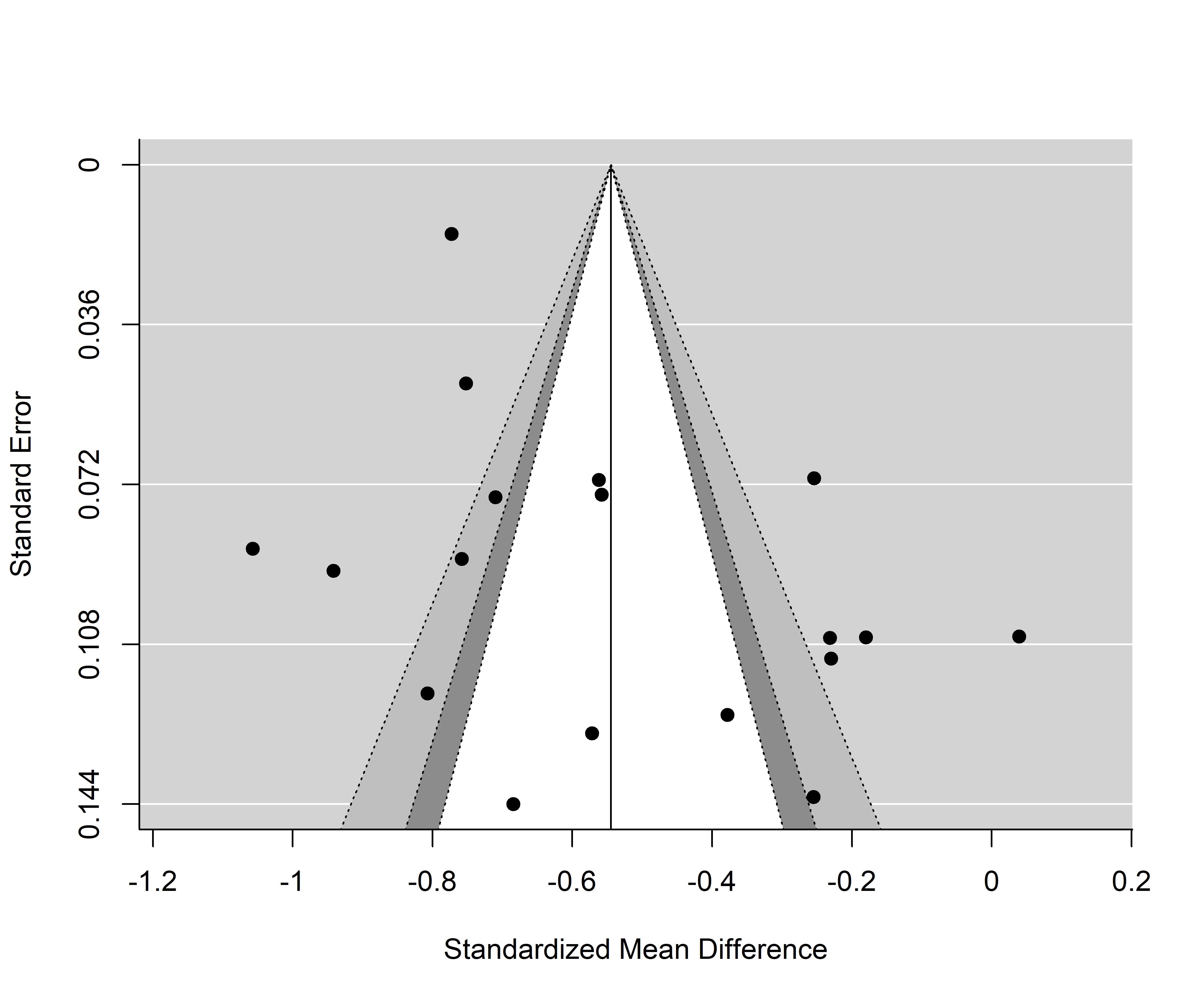

Supplement: Supplementary Figure 1 — Funnel plot to assess publication bias. [file Image1.jpeg]
